# Immunization of Goats with Recombinant Protein 14-3-3 Isoform 2(rHcftt-2) Induced Moderate Protection against *Haemonchus contortus* Challenge

**DOI:** 10.3390/pathogens9010046

**Published:** 2020-01-06

**Authors:** Yongqian Bu, Caiwen Jia, Xiaowei Tian, Kalibixiati Aimulajiang, Muhammad Ali Memon, Ruofeng Yan, Xiaokai Song, Lixin Xu, Xiangrui Li

**Affiliations:** MOE Joint International Research Laboratory of Animal Health and Food Safety, College of Veterinary Medicine, Nanjing Agricultural University, Nanjing 210095, China; 2016207023@njau.edu.cn (Y.B.); 2017807135@njau.edu.cn (C.J.); 2017207011@njau.edu.cn (X.T.); 2017207022@njau.edu.cn (K.A.); 2016207040@njau.edu.cn (M.A.M.); yanruofeng@njau.edu.cn (R.Y.); songxiaokai@njau.edu.cn (X.S.); xulixin@njau.edu.cn (L.X.)

**Keywords:** *Haemonchus contortus*, 14-3-3, immunization, goat

## Abstract

A previous study identified that isoform 2 (Hcftt-2) of the 14-3-3 protein of *Haemonchus contortus* (*H. contortus*) could suppress immune functions of goat peripheral blood mononuclear cells (PBMCs) and might be a potential vaccine target, as neutralization of the protein function may enhance anti-parasite immunity. In this research, the recombinant Hcftt-2 was evaluated for its immunoprotective efficacy against *H. contortus* infection in goats. Five experimental goats were immunized twice with rHcftt-2 along with Freund’s adjuvant. The five immunized goats and five nonimmunized goats (adjuvant only) were challenged with 5000 L3-stage *H. contortus* larvae after 14 days of second immunization. Five nonimmunized and uninfected goats (adjuvant only) were set as the uninfected group. A significant increase in the serum immunoglobin G(IgG) and serum IgA levels were identified in the rHcftt-2 immunized animals. The mean eggs per gram in feces (EPG) and the worm burdens of rHcftt-2 immunized group were reduced by 26.46% (*p* < 0.05) and 32.33%, respectively. In brief, immunization of goats with rHcftt-2 induced moderate protection against *H. contortus* challenge.

## 1. Introduction

Helminth parasites are a worldwide problem, resulting in economic losses in the livestock industry. *Haemonchus contortus* is a highly pathogenic blood-sucking gastrointestinal nematode of the abomasum of small ruminants. Infections with this nematode can cause anemia, weight loss, and even death [1,2,3,4]. Current methods of prevention and control depend on the use of anthelmintics [3]. However, the appearance of anthelmintic-resistant strains, the environmental pollution, and the toxic residues in animal products has led to the urgent need for the development of more effective vaccines [5].

Until now, many native and recombinant proteins have been investigated in different immunization trials against *H. contortus* and most cases demonstrated variable reduction in fecal egg output and worm burdens [3,6,7,8,9]. DNA vaccines have also been tested and showed partial protection against *H. contortus* [10,11,12]. Although a huge progress in vaccine research has occurred, only one commercial vaccine called Barbervax (Moredun Research Institute, Penicuik, Mid-Lothian, UK) is widely used in Australia [13]. Unfortunately, some ethical considerations exist limiting the use of infected animals as a source of isolating the worms and consequently harvesting the native proteins (H11 and H-gal-GP protein complexes). To date, bio-engineering technology has supported the production of many parasite antigens in a heterologous system with a high level of purity. The need to identify more vaccine targets for practical application has been emphasized by some recombinant antigens being unable to induce the same protection as the native antigens in many cases [6,14].

The 14-3-3 proteins are crucial regulators of intracellular signaling pathways, involved in various biological processes, including cell proliferation, growth, and apoptosis [15]. They are a family of highly conserved, acidic proteins expressed in all eukaryotic cells [16]. Different 14-3-3 isoforms have unique and fundamental roles [17]. 14-3-3 proteins have also been identified in many kinds of parasites. In-depth studies have been performed in *Toxoplasma gondii* [17], *Echinococcus* [18], *Schistosoma mansoni* [19], *Eimeria* [20], and *H. contortus* [21]. In a previous study on *H. contortus*, 14-3-3 protein was found to be released as an excretory and secretory protein that could interact with goat peripheral blood mononuclear cells (PBMCs). *H. contortus* 14-3-3 isoform 2 (Hcftt-2) displayed important suppressive regulatory effects on the goat PBMCs [21]. In theory, if immunosuppressive molecules are delivered to the host, they induce a significant circulating antibody response. The antibodies neutralize the molecule and thereby subvert the suppressive functions, which are beneficial for the survival of the parasite [22]. Several immunomodulatory molecules have been tested as vaccine candidates. The recombinant pattern of three molecules, apyrase, macrophage migration inhibitory factor, and transforming growth factor-β(TGF-β) homologue, were emulsified with Quil A as a cocktail vaccine. The results showed a successful immune protect against *Teladorsagia circumcincta* in sheep [23]; Recombinant galectin can suppress T cell proliferations; however, vaccination of goats with recombinant galectin antigen induces partial protection against *H. contortus* infection [6,24]. Given the important roles attributed to modulate the immune system of the host, Hcftt-2 could be considered as a vaccine candidate against *H. contortus*. To date, 14-3-3 proteins as vaccine candidates have been tested in *T. gondii* and *Schistosoma japonicum* and have shown protective efficacy [25,26]. So, in this study, we examined the immunoprophylactic efficacy of the recombinant Hcftt-2 against *H. contortus* for the first time in goat.

## 2. Results

### 2.1. Sodium Dodecyl Sulfate–Polyacrylamide Gel Electrophoresis (SDS-PAGE) Profile of rHcftt-2 and Immunoblot

**Figure 1:**
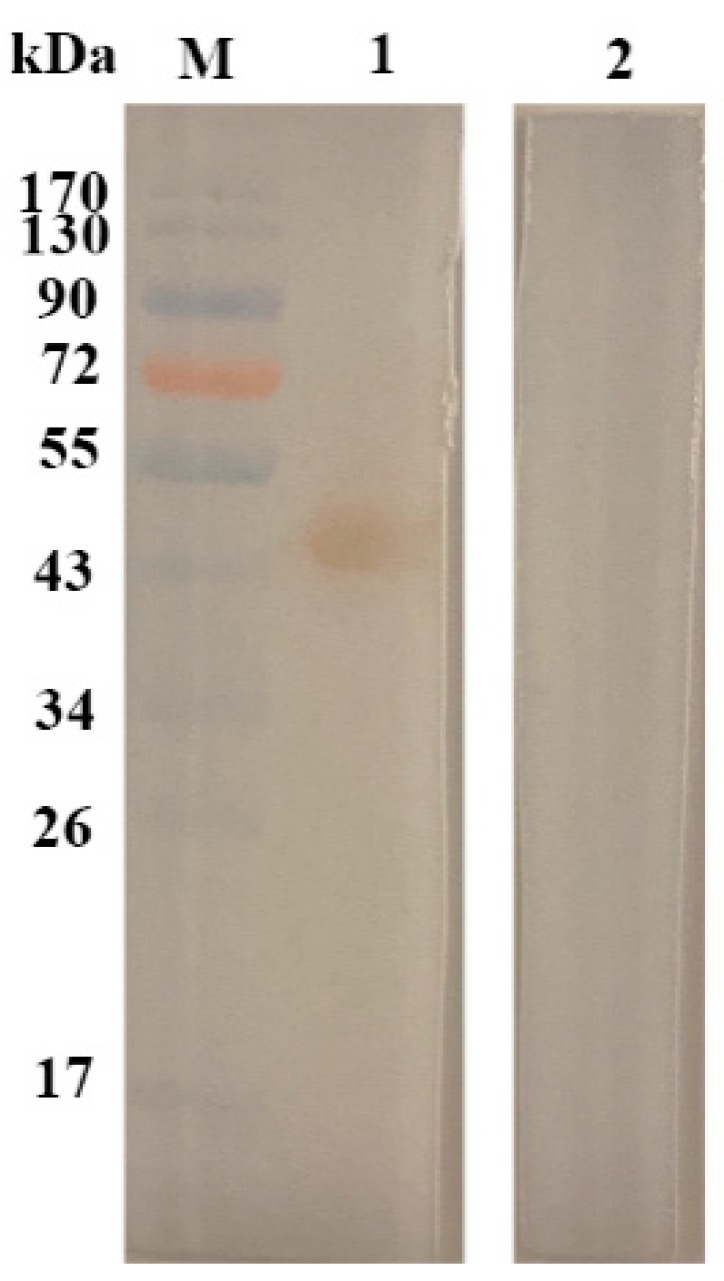
Immuoblot analysis of rHcftt-2 using goats’ sera. Line1: rHcftt-2 were detected by the sera collected from *H. conturtus* infected goats. Line 2: rHcftt-2 were checked by the sera collected form normal goats. Line M: the protein marker.

The expressed product and the purified protein were detected using SDS-PAGE after staining with Coomassie brilliant blue. The target protein was purified from the cell supernatants with the molecular weight of 48 kDa. Sera from infected goats revealed the presence of sero-reactive bands at 48 kDa. Immunoblot results showed that rHcftt-2 could be detected by the sera from infected goats (Figure 1).

### 2.2. Fecal Eggs Counts and Worm Burdens

**Figure 2:**
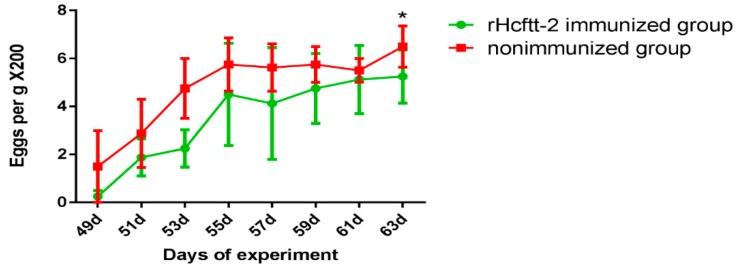
Dynamics of fecal egg counts of immunized and nonimmunized groups. Eggs per gram (EPG) was expressed as mean ± standard error of the mean (SEM). Goats immunized rHcftt-2 and challenged/rHcftt-2 immunized group (green); goats unvaccinated but challenged/nonimmunized group (red). Eggs were detected since day 49 of the experiment (21 days after challenge). (* *p* < 0.05).

The fecal output of *H. contortus* eggs (egg per gram, EPG) in each group after challenge is shown in Figure 2. The results indicated that the egg shedding began on day 49 of the experiment (21 days post-larval challenge) and increased gradually to a peak on day 63 (35 days post-challenge). In contrast, no eggs were found from the nonimmunized and uninfected goats during the study. The reduction in average EPG was 26.46% compared with the control animals (*p* < 0.05). 

**Figure 3:**
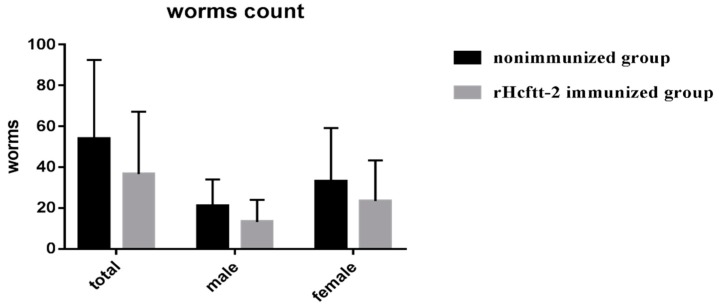
The number of female and male worm counts in different groups. Results were showed as mean ± SEM, Goats immunized rHcftt-2 and challenged/rHcftt-2 immunized group; goats unvaccinated but challenged/nonimmunized group.

Similarly, the worm burden in group rHcftt-2 immunized animals was reduced compared with the nonimmunized and uninfected group. The female, male and total worm reductions were 37.14%, 29.09%, 32.22%, respectively (Figure 3). The worms found in each group are showed in the Supplementary Table S1. However, all differences in worm reductions were not statistically significant. 

### 2.3. Serum Antibody Response

**Figure 4:**
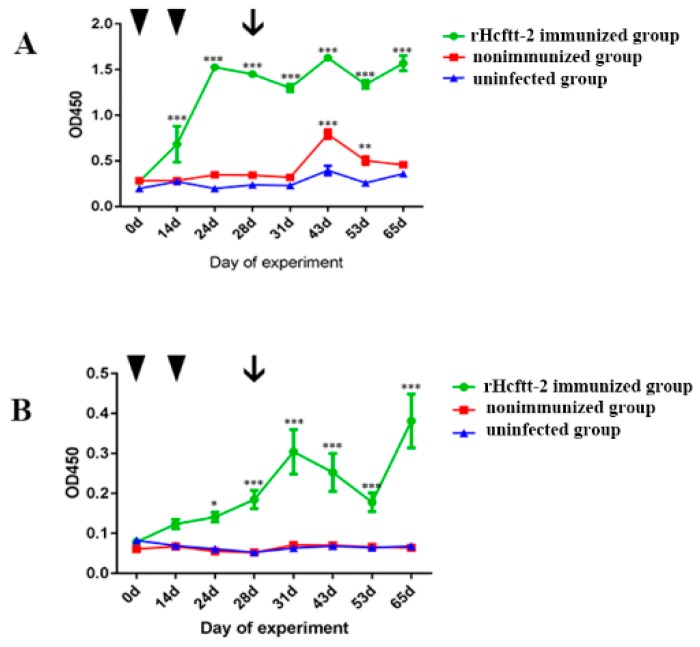
Levels of specific serum IgG (**A**) and IgA (**B**) in the experiments. Goats immunized with rHcftt-2 and challenge/rHcftt-2 immunized group (green); goats uninfected and unimmunized/uninfected group (blue); goats unvaccinated but challenged/nonimmunized group (red). The results were expressed as mean ± SEM. The triangle indicated the vaccinated time and the arrow indicated the challenged time. (* *p* < 0.05, ** *p* < 0.01, *** *p* < 0.001).

The serum specific IgG for rHcftt-2 of groups 1, 2, and 3 is plotted in Figure 4A. Group mean (± standard error of the mean (SEM)) serum IgG levels increased after the first immunization in the rHcftt-2 immunized group (group 1) and reached a peak at day 24. These levels remained significantly high until the end of the study (*p* < 0.05). The specific IgG levels of group 2 were significantly higher than the uninfected group (group 3) at days 43 and 53. In contrast, the specific IgG levels of the uninfected group remained low throughout the trial.

Similarly, the specific serum IgA for rHcftt-2 levels was significantly higher (*p* < 0.05) in group 1 at day 24 compared with those of the nonimmunized group and uninfected group (groups 2 and 3, respectively; Figure 4B). A low level of specific IgA response was detected in sera collected from the two groups throughout this study. 

### 2.4. Concentration of Serum Cytokines

**Figure 5:**
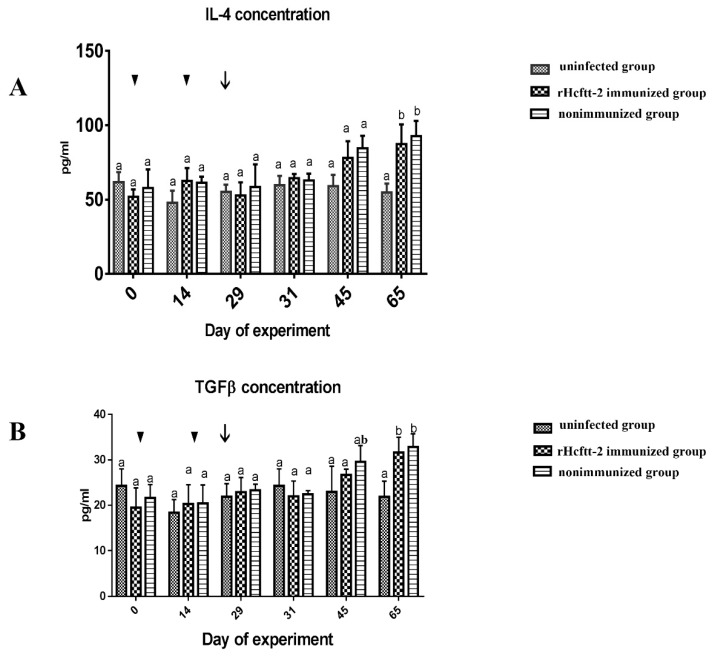
Determination of serum cytokines. The results are shown as mean ± SEM. (**A**) IL-4 concentration; (**B**) TGF-β concentration. Triangle, vaccination time; arrow, challenged time. Values bearing a different superscript letter (a,b) differ significantly from one another (*p* < 0.05), the column with a is significantly different from the column with b. The column with ab is not different from the column with b.

We found no statistical change in serum interleukin-4 (IL-4) concentration observed before the L3 larval challenge in all groups. Figure 5A indicates that the IL-4 concentration in the rHcftt-2 immunized group (group 1) increased after challenge and distinctly rose on day 65 (*p* < 0.05). The IL-4 concentration in the nonimmunized group (group 2) showed the same change as the immunized group (group 1). However, the concentration remained constant during the whole experiment in the uninfected group (group 3). The same result was observed during evaluation of serum TGF-β concentration. TGF-β concentrations in the immunized and nonimmunized groups (groups 1 and 2, respectively) were significantly higher than the uninfected group (group 3) at day 65 (*p* < 0.05), and we observed no obvious difference in TGF-β concentration between the immunized group (group 1) and nonimmunized group (group 2; Figure 5B).No difference was recorded for IL-10, IL-17, and Interferon-γ (IFN-γ) concentrations in all groups during the trial. 

### 2.5. Differential Cell Counts

**Figure 6:**
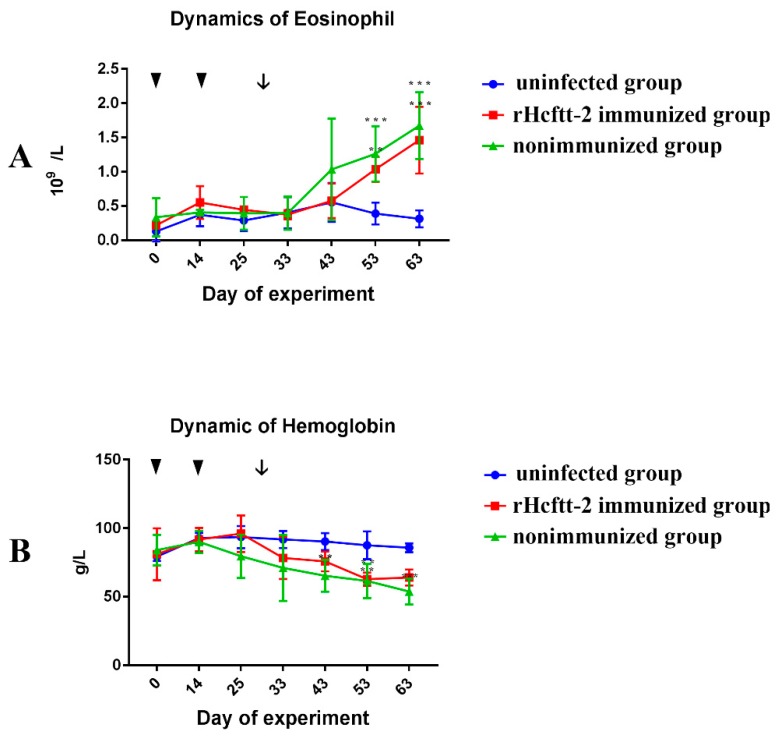
Dynamics of Eosinophill (**A**) and Hemoglobin (**B**). Results were expressed as mean ± SEM. Goats immunized rHcftt-2 and challenged/rHcftt-2 immunized group (red); goats unvaccinated but challenged/nonimmunized group (green); goats uninfected and nonimmunized/uninfected group (blue). The triangle indicated the vaccinated time and the arrow indicated the challenged time. (** *p* < 0.01, *** *p* < 0.001).

The eosinophils dynamics in each group are plotted in Figure 6A. The eosinophil counts of immunized group (group 1) and nonimmunized group (group 2) were enhanced after L3 challenge and were significantly higher (*p* < 0.05) compared to that of the uninfected group (group 3) at day 63.

The hemoglobin of the immunized group (group 1) and nonimmunized group (group 2) decreased after L3 challenge. At day 43, the hemoglobin concentration of nonimmunized group was significantly lower than the uninfected group (group 3). Although the hemoglobin of immunized group (group 1) was lower than the uninfected group (group 3), the difference was not significant (Figure 6B). 

We identified no significant changes in the number of lymphocytes and monocytes in all groups during the trial.

## 3. Discussion

In previous studies, *H. contortus* 14-3-3 was identified as an excretory and secretory protein that could bind to goat PBMCs [27]. A subsequent study showed that the *H. contortus* 14-3-3 isoform 2(Hcftt2) might mainly play an immunosuppressive role in the infection of *H. contortus* [21]. Protective immunity induced by 14-3-3 protein from other parasites like *Toxoplasma gondii* [25], *Trichinella spiralis* [28], *Echinococcus granulosus* [29], and *Schistosoma mansoni* [30] has been evaluated. In this study, rHcftt2 was evaluated for its protective efficacy against *H. contortus* infection. 

Fecal egg output in *H. contortus* infections is probably the best in vivo phenotypic marker of resistance or protection [3,9]. In this research, goats immunized with rHcftt2 showed a significant 26.46% reduction in egg output, and the same vaccinated animals displayed a 32.22% reduction in the amount of adult *H. contortus* collected from the abomasa compared with the positive control group at the end of the trial. The female adult reduction was higher than the male reduction, most likely indicating the higher blood intake of the female worms [8]. Unfortunately, the reductions of adult worms were not statistically significant, probably due to the presence of nonresponsive animals in each group and the sampling size. A similar but slightly higher degree of protection was reported in recombinant galectin vaccinated goats [6].

The result of immunoblotting showed that the sera from infected animals could recognize the recombinant Hcftt-2 protein. This indicated that the sera from infected animals contain the IgG against the protein. Therefore, the native 14-3-3 of *H. contortus* could be exposed to the goat immune system during infection. This also proved that 14-3-3 protein is released as an excretory/secretory protein.

In this trial, the rHcfft-2 immunized group showed higher levels of serum IgG and IgA compared with control animals. Similar results were described in vaccination trials in which immunization was with native or recombinant helminth antigens [6,10,31]. In the nonimmunized group after the infection, the specific serum IgA was not detected; the specific serum IgG was not significant but was higher than the uninfected group at day 65. These phenomena seem to indicate that Hcftt-2 could not be secreted for a long time or that the antigenicity is weak. A similar result was reported in other research [3]. As reported previously, antibodies may play a role in the immunoprotection against *H. contortus* [10,32,33]. Considering the immunosuppressive functions of *H. contortus* 14-3-3, the specific antibodies developed after immunization could have a neutralizing effect on *H. contortus* 14-3-3, which is excreted and secreted during the infection. Consequently, the nematode was exposed to the developing protective immune system. 

*H. contortus* infection affecting and reducing the hemoglobin level of the host is well known. In this study, this was also observed in the rHcftt-2 immunized and positive control groups after challenge. The hemoglobin level of the rHcftt-2 immunized group was higher than in the positive control group, but still lower than the negative control group. The mean hemoglobin value of the rHcftt-2 immunized group was still in the normal physiological range of this local breed (62–135 g/L). This suggests that immunization with rHcftt-2 could alleviate the hemoglobin loss in hosts after challenge. 

In previous studies, sheep infected with *H. contortus* elicited a T-helper cell type 2 response, given increased IL-4 both at the gene and the protein levels [34,35,36]. Upregulation of IL-4 in *H. contortus* infection has been attributed to generating specific IgE [37,38]. TGF-β is a well-known cytokine that plays an immunosuppressive role in the immune system. TGF-β is secreted by regulatory T(Treg) cells, down-regulating the immune system after activation in response to allergens and pathogens [39]. In a previous study, mice serum levels of TGF-β were found to be increased after infection of *Fasciola hepatica* [40]. In this study, the concentrations of serum IL-4 and TGF-β in the immunized group and the infection group were both increased after L3 challenge, the concentrations in the nonimmunized and uninfected group (group 3) were still lower even when they were injected with adjuvant. We found no differences between the immunized group (group 1) and nonimmunized group (group 2). Similar results for cytokines were observed in other trials [6,10]. The concentrations of IFN-γ and IL-17 in the immunized group were unchanged during the trial, and immunization with rHcftt-2 appears to be unable to induce Th1 immune response and Th17 proliferation. We found no change in IL-10 concentration, which is contrary to the change in TGF-β in this trial. These results reflect the difficulty of development of the vaccines, and more immunoregulatory mechanisms need be researched.

Eosinophilia is a central feature of the host response to helminth infection [41]. Eosinophils release cationic proteins with significant cytotoxic activity that damages the parasites. In previous studies, eosinophils were found to adhere to the L3 larvae and reduce their establishment potential in sheep [42]. In this research, the peripheral eosinophils count of the immunized group (group 1) and the nonimmunized group (group 2) were higher than the uninfected group (group 3). However, the difference between the immunized and nonimmunized groups was not significant. This could perhaps be related to the cytokines. Various cytokines and immune cells interact to maintain the balance between host and the parasite. Similar results were also obtained in a previous research [11]. 

In this study, the Hcfft-2 was chosen for vaccine development due to its immunosuppressive characteristics. Specific antibodies may neutralize the immunosuppressive effect of antigens. Even though several attempts were successful, our results do not seem to meet expectations. In this process, the rHcftt-2 may still exert a certain immunosuppressive function on the host, even when the specific antibodies increased after immunization. Native Hcftt-2 was possibly limitedly released, and the acquired antibodies in the immunized goats were limited in neutralizing the protein after infection. A normal level of hemoglobin in the immunized group suggested that immunization with rHcftt-2 plays a positive role in preventing anemia. The increased in IL-4 and eosinophils in the immunized group may indicate an activated Th2 response. A type of regulatory cytokine, TGF-β, also increased after infection. This could negatively regulate the immune response to the nematode. These conflicting changes are perhaps regulated by the nematode products after infection. Therefore, research of the regulatory mechanism is worth studying further.

## 4. Material and Methods

### 4.1. Infective Larvae for Challenge

The *H. contortus* strain used in this trial was originally obtained from Nanjing, Jiangsu Province, China and it was maintained by serial passage in goats. Feces were collected every day and cultured at 28 °C. The harvested L3 used for challenge were stored in distilled water at 4 °C and kept not more than 1 month.

### 4.2. Expression and Purification of rHcftt-2

The recombinant expression vector pET32a-Hcftt-2 was constructed in a previous experiment, and was stored at −80 °C [21]. The plasmid was transformed in *Escherichia coli* BL21 (DE3). A positive clone was picked out and cultured in Luria-Bertani (LB)-ampicillin medium at 37 °C until the optical density at 600 nm was 0.6. Protein was harvested after another 5 h of incubation after adding isopropyl B-D-thiogalactopyranoside (IPTG). Bacteria were lysed by sonication, and the recombinant fusion protein was purified using a Ni-Nitrilotriacetic acid (NTA) affinity column (HiTrap, GE, Uppsala, Uppsala, Sweden). After removing the imidazole in phosphate-buffered saline (PBS), the endotoxins were also removed using ToxinEraser^TM^ Kit (GeneScript, Nanjing, Jiangsu, China). The purified recombinant protein was checked in 12% SDS-PAGE, and the concentration was estimated using a bicinchoninic acid (BCA) protein assay kit (Pierce, Dallas, TX, USA).

### 4.3. Immunoblot Analysis of rHcftt-2 Using Goats Sera

The purified recombinant protein was separated on a 12% SDS-PAGE gel and then transferred to a polyvinylidene fluoride (PVDF) membrane (Millipore, Tullagreen, Carrigtwohill, Ireland) using semi dry-blot electrophoresis transfer cell (BioRad, Hercules, CA, USA). After being blocked with 5% skimmed milk in PBS–Tween (PBST) at 4 °C overnight, the membranes were incubated with the pooled sera collected from goats infected with *H. contortus* (1:50 dilution) for 1 h at 37 °C. The pooled sera from goats free of *H. contortus* infection were also used as the control. Then, the membrane was washed with PBST for 5 times and incubated with rabbit anti-goat IgG-horseradish peroxidase (HRP,1:5000 dilution; Bioworld, Nanjing, Jiangsu, China) for 1 h at 37 °C. After being washed 5 times, a signal was detected using a 3,3-diaminobenzidine-tetrahydrochloride (DAB) kit (Beyotime Biotechnology, Shanghai, China).

### 4.4. Animals and Vaccination Protocol

Fifteen female local breed goats were purchased from Luhe District (Nanjing, China). These experimental animals were reared strictly from birth to avoid parasitic infection. They were transported to the animal house when they were 6–7 months old. At their arrival, the parasitological examination showed no helminth infections. All the animals were reared in covered pens under the clean conditions designed to preclude nematode infections. They were fed with commercial pellet food (Qinglongshan, Nanjing, Jiangsu, China). Water and mineralized salt bricks were available ad libitum. After 45 days of adapted feeding, all the goats were randomly allocated into three groups of 5 goats each. Group 1 (n = 5) consisted of goats immunized with rHcftt-2 and challenged with *H. contortus*. Group 2 (n = 5) consisted of goats nonimmunized but experimentally infected with *H. contortus.* Group 3 (n = 5) consisted of goats nonimmunized and uninfected. The goats in group 1 were injected with 500 μg rHcftt-2 on day 1. The protein was emulsified with complete Freund’s adjuvant (Sigma, St. Louis, MO, USA). On day 14, the goats were immunized with the same dose of rHcftt-2 emulsified with incomplete Freund’s adjuvant (Sigma, St. Louis, Mo, USA). The goats in groups 2, 3 were administered an injection of 1 mL PBS and Freund’s adjuvant, following the same protocol of inoculation for group 1. On day 28, two weeks after the second injection, every goat in groups 1 and 2 was infected with 5000 *H. contortus* L3. All the animals were sacrificed at day 66, 38 days after being challenged with *H. contortus.*

The animal experiment was approved by the laboratory animal center of Nanjing agricultural university. All efforts guaranteed the animals’ welfare and minimized animal suffering. The animals were treated according to the Measures for the Management of Experimental Animals in Jiangsu province (Decree No.45 of the people’s Government of Jiangsu Province).

### 4.5. Parasitological Measurements

Beginning on day 46, 18 days after challenge, fresh feces were collected directly from the rectum every day until the egg shedding was detected and every two days afterwards. Fecal egg counts were determined immediately after the sample was collected using the Mc-Master method [43], and the results are presented as eggs per gram of feces (EPG). 

The ligated abomasa were removed from the goats after they were sacrificed. After washing the opened abomasa with clean water thoroughly, all the contents were placed in corresponding basins. The total numbers of adult worms were counted carefully and sorted according to sex.

### 4.6. Differential Cell Count 

To acquire more details during the trial, the blood samples with anticoagulant were collected on days 0, 14, 25, 33, 43, 53, and 63. The hemoglobin and cell counts were carried out by automated hematology analyzer with five classifications (mindray BC-5000 Vet, Shengzhen, China).

### 4.7. Detection of Antibodies in Serum

To confirm the presence of specific antibodies (IgG) against rHcftt-2, indirect ELISA was used. Ninety-six-well microplates were coated with 100 µL of rHcftt-2 (1 µg/mL) at 4 °C overnight. After being washed with PBST, plates were blocked with 5% (w/v) skimmed milk in PBS for 1 h at 37 °C. Individual sera were added at dilution 1:1000, and 100 µL was added to each well and incubated for 1 h at 37 °C. Then, the plate was washed 5 times, and the HRP-conjugated anti-goat IgG (1:5000, Bioworld, Nanjing, Jiangsu, China) was used as the second antibody and added to each well for 1h incubation at 37 °C. Following 5 washings TMB-peroxidase (Beyotime Biotechnonogy, Shanghai, China) was added to each well. After 20 min incubation in the dark, the reaction was stopped using 2 mol/L sulfuric acid, and the optical density was read with a microplate ELISA reader at 450 nm (Multiskan FC, ThermoFisher, Vantaa, Finland). The concentrations of serum IgA were measured using the same method, but the serum samples were diluted to 1:100 as the first antibody, and the HRP-conjugated anti-goat IgA (Abcam, 1:5000, Shanghai, China) was used as the second antibody.

### 4.8. Determination of Serum Cytokine Concentration

At days 0, 4, 29, 31, 45, and 66 of the experiment, the serum of each goat was collected, and the concentration of certain cytokines was detected using the Goat cytokine ELISA Quantitation Kits (catalogue numbers: IL-4 ml9025539, TGF-β ml2025653, IFN-γ ml6027464, IL-10 ml6010007, IL-17 ml9036682,Enzyme-Linked, Shanghai, China). All the operations were carried out according to the manufacturer’s instructions. 

### 4.9. Statistical Analysis

Statistical analysis was performed using SPSS statistical package (SPSS for Windows 17, SPSS INC., Chicago, IL, USA). Differences between groups were tested by one-way ANOVA and Duncan’s test. 

## 5. Conclusions

In conclusion, immunization of goats with rHcftt-2 induced modest protection against *H. contortus* infection in goats. Given the slight reductions in parasitological parameters in this trial, different candidate antigens should be explored in the future.

## Supplementary Materials

The following are available online at https://www.mdpi.com/2076-0817/9/1/46/s1, Table S1: *Haemonchus contortus* burden in the abomasum of the goats at the end of experiment.
