# Supplementary material for: Immunization of Goats with Recombinant Protein 14-3-3 Isoform 2(rHcftt-2) Induced Moderate Protection against Haemonchus contortus Challenge
Source: Pathogens. 2020 Jan 6;9(1):46. doi: 10.3390/pathogens9010046 (PMC7168593; doi:10.3390/pathogens9010046)
Supplement: Supplementary file 1 [file pathogens-09-00046-s001.pdf]

pathogens

MDPI

Table S1. *Haemonchus contortus* burden in the abomasum of the goats at the end of experiment

Group	No. Goats	No.female worms	No.male worms	Total
Immunized	1	54	30	84
	2	28	12	40
	3	15	10	25
	4	20	14	34
	5	0	0	0
Mean±SEM		23.4±8.908	13.2±4.841	36.6±13.67
Nonimmunized	6	28	22	50
	7	72	35	107
	8	40	27	67
	9	25	21	46
	10	0	0	0
Mean±SEM		33±11.72	21±5.805	54±17.28
